# Synthesis, crystal structure and Hirshfeld surface analysis of 3-(4,4-dimethyl-2,3,4,5-tetra­hydro-1*H*-1,5-benzodiazepin-2-yl­idene)-6-methyl-3,4-di­hydro-2*H*-pyran-2,4-dione

**DOI:** 10.1107/S2056989019000689

**Published:** 2019-01-18

**Authors:** Mohamed Samba, Mohamed Said Minnih, Tuncer Hökelek, Manpreet Kaur, Jerry P. Jasinski, Nada Kheira Sebbar, El Mokhtar Essassi

**Affiliations:** a Laboratoire de Chimie Organique Hétérocyclique URAC 21, Pôle de Compétence Pharmacochimie, Av. Ibn Battouta, BP 1014, Faculté des Sciences, Université Mohammed V, Rabat, Morocco; b Unité de Chimie Moléculaire et Environnement, Université de Sciences, de Technologie et de Médecine, BP 5026, Nouakchott, Mauritanie, Morocco; c Department of Physics, Hacettepe University, 06800 Beytepe, Ankara, Turkey; d Department of Studies in Chemistry, University of Mysore, Manasagangotri, Mysore 570 006, India; e Department of Chemistry, Keene State College, 229 Main Street, Keene, NH 03435-2001, USA; f Laboratoire de Chimie Bioorganique Appliquée, Faculté des Sciences, Université Ibn Zohr, Agadir, Morocco; g Moroccan Foundation for Advanced Science, Innovation and Research (MASCIR), Rabat, Morocco

**Keywords:** crystal structure, benzodiazepine, hydrogen bond, Hirshfeld surface

## Abstract

The title compound, C_17_H_18_N_2_O_3_, is constructed from a benzodiazepine ring system linked to a pendant di­hydro­pyran ring, where the benzene and pendant di­hydro­pyran rings are oriented at a dihedral angle of 15.14 (4)°. Intra­molecular N—H_Diazp_⋯O_Dhydp_ and C—H_Diazp_⋯O_Dhydp_ (Diazp = diazepine and Dhydp = di­hydro­pyran) hydrogen bonds link the seven-membered diazepine ring to the pendant di­hydro­pyran ring, enclosing *S*(6) ring motifs. In the crystal, N—H_Diazp_⋯O_Dhydp_ hydrogen bonds link the mol­ecules into infinite chains along [10]. These chains are further linked *via* C—H_Bnz_⋯O_Dhydp_, C—H_Dhydp_⋯O_Dhydp_ and C—H_Mth_⋯O_Dhydp_ (Bnz = benzene and Mth = meth­yl) hydrogen bonds, forming a three-dimensional network. The observed weak C—H_Diazp_ ⋯ π inter­action may further stabilize the structure. Hirshfeld surface analysis of the crystal structure indicates that the most important contributions for the crystal packing are from H⋯H (51.1%), H⋯C/C⋯H (25.3%) and H⋯O/O⋯H (20.3%) inter­actions. Hydrogen bonding and van der Waals inter­actions are the dominant inter­actions in the crystal packing.

## Abstract 2

The title compound is built up from the benzodiazepine ring system linked to the pyridyl and pendant di­hydro­pyran rings. In the crystal, N—H⋯O and C—H⋯O hydrogen bonds link the mol­ecules into a three-dimensional network. A weak C—H ⋯ π inter­action is also observed.

## Chemical context   

Derivatives of 1,5-benzodiazepines have attracted considerable attention from researchers because of their bioactive and pharmaceutical properties. Many members of this family are widely used as anti­convulsant, anti-anxiety, anti-seizure, analgesic, sedative, anti­depressive and hypnotic or anti-inflammatory agents (Kudo, 1982 ▸; Roma *et al.*, 1991 ▸; Rajarao *et al.*, 2007 ▸; Kumar & Joshi, 2007 ▸; Guerrini *et al.*, 2006 ▸). Diversely substituted 1,5-benzodiazepines and their derivatives embedded with a variety of functional groups are important biological agents that have been the subject of a significant amount of research activity (Kotyatkina *et al.*, 2001 ▸; Fruscella *et al.*, 2001 ▸; Zellou *et al.*, 1998*a* ▸,*b* ▸). Over the last decade, biological inter­est in 1,5-benzodiazepines has extended to include their use as anti­bacterial and anti­fungal agents (Kalkhambkar *et al.*, 2008 ▸; Smith *et al.*, 1998 ▸). Benzodiazepine derivatives also find commercial use as dyes for acrylic fibers and as inter­mediates in the synthesis of several heterocyclic systems (Essassi & Salem, 1985 ▸; Minnih *et al.*, 2014 ▸; Rida *et al.*, 2018 ▸). The search for new heterocyclic systems including the 1,5-benzodiazepine moiety for bio­logical activities is therefore of much current importance (Essassi & Salem, 1985 ▸; Dardouri *et al.*, 2011 ▸; Chkirate *et al.*, 2018 ▸; Keita *et al.*, 2003 ▸; Jabli *et al.*, 2009 ▸). In this context, we synthesized the title compound namely 3-(4,4-dimethyl-2,3,4,5-tetra­hydro-1*H*-1,5-benzodiazepin-2-yl­idene)-6-methyl-3,4-di­hydro-2*H*-pyran-2,4-dione by reacting de­hydro­acetic acid and *o*-phenyl­enedi­amine in ethanol, and we report here the synthesis, the mol­ecular and crystal structures along with the Hirshfeld surface analysis.
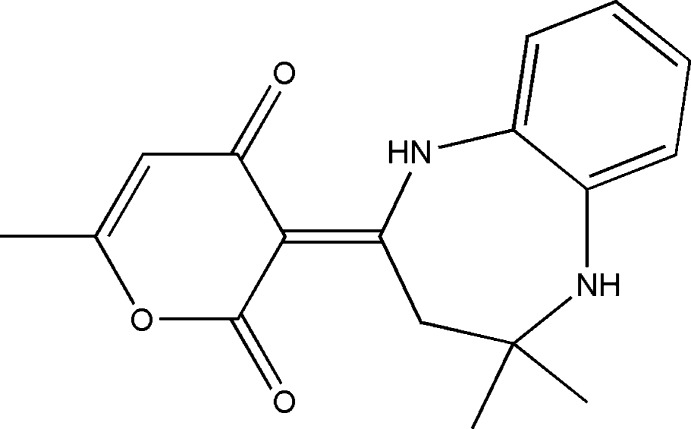


## Structural commentary   

**Figure 1:**
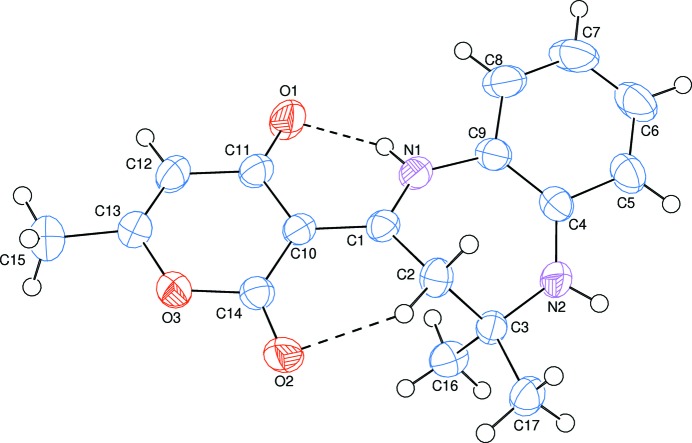
The mol­ecular structure of the title compound with the atom-numbering scheme. Displacement ellipsoids are drawn at the 50% probability level. N—H_Diazp_⋯O_Dhydp_ and C—H_Diazp_⋯O_Dhydp_ (Diazp = diazepine and Dhydp = di­hydro­pyran) hydrogen bonds are shown as dashed lines.

The title compound, (I), is built up from a benzodiazepine ring system linked to a pendant di­hydro­pyran ring, (*C*: O3/C10–C14) (Fig. 1 ▸). Ring *C* is planar within 0.0381 (13) Å (for atom C10), and is oriented at a dihedral angle of 15.14 (4)° with respect to the benzene (*B*: C4–C9) ring. A puckering analysis of the seven-membered diazepine ring (*A*: N1/N2/C1–C4/C9) gave the parameters *Q*_T_ = 0.6874 (14), *q*_2_ = 0.5903 (14), *q*_3_ = 0.3523 (14) Å, φ_2_ = 352.88 (14), φ_3_ = 245.8 (2)°. In ring *A*, the N1—C1—C2 [117.10 (2)°], C1—C2—C3 [114.17 (11)°], C3—N2—C4 [128.91 (11)°], N2—C4—C9 [127.38 (12)°], C4—C9—N1 [126.21 (12)°] and C9—N1—C1 [130.71 (12)°] bond angles are enlarged, while the C2—C3—N2 [108.97 (11)°] bond angle is narrowed, when compared with the corresponding values in the seven-membered diazepine ring in the closely related compound, 3,4-di­hydro-2-(2,4-dioxo-6-methyl­pyran-3-yl­id­ene)-4-(4-pyridin-4-yl)-1,5-benzo- diazepine, (II), where the pendant di­hydro­pyran ring is not planar (El Ghayati *et al.*, 2019 ▸). On the other hand, the N1—C1 [1.3168 (18) Å], N2—C3 [1.4542 (17) Å], N2—C4 [1.3584 (19) Å], C1—C2 [1.4907 (18) Å] and C2—C3 [1.5444 (18) Å] bond lengths in ring *A* in (I) may be compared with the corresponding values of N2—C9 [1.3206 (18) Å], N1—C7 [1.4648 (18) Å], N1—C6 [1.3996 (18) Å], C8—C9 [1.5020 (18) Å] and C7—C8 [1.5291 (19) Å] in (II).

In the mol­ecule of (I), N—H_Diazp_⋯O_Dhydp_ and C—H_Diazp_⋯O_Dhydp_ (Diazp = diazepine and Dhydp = di­hydro­pyran) hydrogen bonds (Table 1 ▸) link the seven-membered diazepine ring *A* to the pendant di­hydro­pyran ring *C*, enclosing *S*(6) ring motifs (Fig. 1 ▸).

## Supra­molecular features   

**Figure 2:**
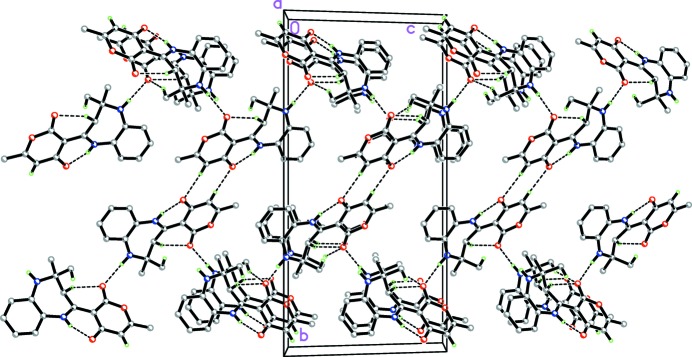
A partial packing diagram viewed along the *a* axis. N—H⋯O and C—H⋯O hydrogen bonds are shown as dashed lines. H atoms not involved in these inter­actions have been omitted for clarity.

In the crystal, N—H_Diazp_⋯O_Dhydp_ hydrogen bonds (Table 1 ▸) link the mol­ecules into infinite chains along [10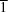]. These chains are further linked *via* C—H_Bnz_⋯O_Dhydp_, C—H_Dhydp_⋯O_Dhydp_ and C—H_Mth_⋯O_Dhydp_ (Bnz = benzene and Mth = meth­yl) hydrogen bonds (Table 1 ▸), forming a three-dimensional network (Fig. 2 ▸). The weak C—H_Diazp_⋯π inter­action (Table 1 ▸) may further stabilize the structure.

## Hirshfeld surface analysis   

**Figure 3:**
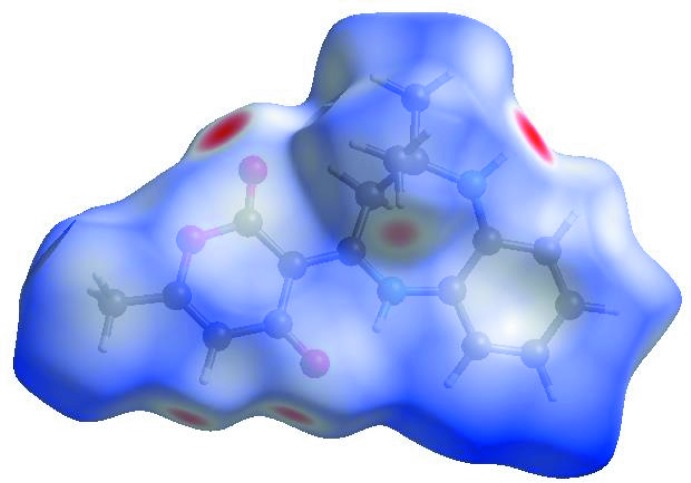
View of the three-dimensional Hirshfeld surface of the title compound plotted over *d*_norm_ in the range −0.4583 to 1.6329 a.u.

**Figure 4:**
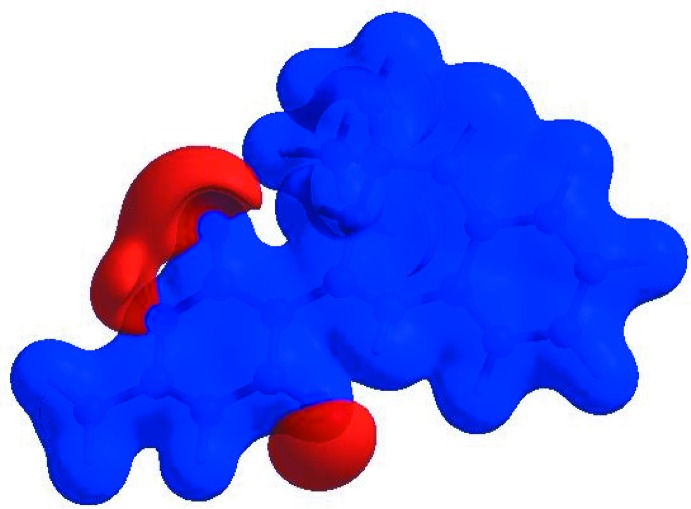
View of the three-dimensional Hirshfeld surface of the title compound plotted over electrostatic potential energy in the range −0.0500 to 0.0500 a.u. using the STO-3 G basis set at the Hartree–Fock level of theory. Hydrogen-bond donors and acceptors are shown as blue and red regions around the atoms, corresponding to positive and negative potentials, respectively.

**Figure 5:**
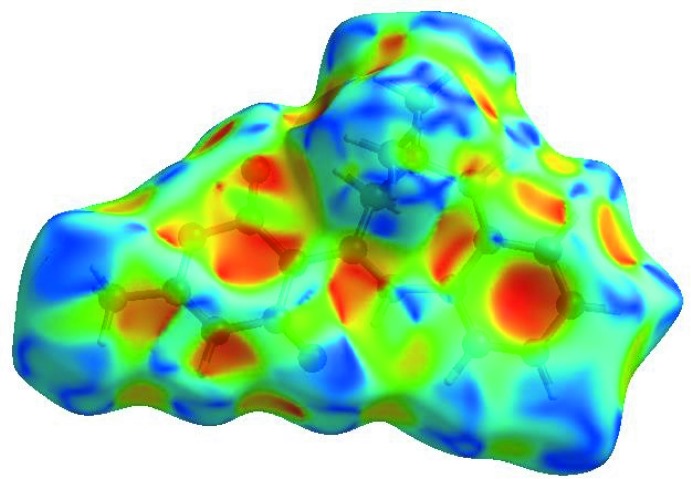
Hirshfeld surface of the title compound plotted over shape-index.

In order to visualize the inter­molecular inter­actions in the crystal of the title compound, a Hirshfeld surface (HS) analysis (Hirshfeld, 1977 ▸; Spackman & Jayatilaka, 2009 ▸) was carried out by using *CrystalExplorer17.5* (Turner *et al.*, 2017 ▸). In the HS plotted over *d*_norm_ (Fig. 3 ▸), the white surface indicates contacts with distances equal to the sum of van der Waals radii, and the red and blue colours indicate distances shorter (in close contact) or longer (distinct contact) than the van der Waals radii, respectively (Venkatesan *et al.*, 2016 ▸). The bright-red spots appearing near atoms O1 and O2 and hydrogen atoms H2, H12 and H16*A* indicate their roles as the respective donors and/or acceptors in the dominant N—H⋯O and C—H⋯O hydrogen bonds (Table 1 ▸); they also appear as blue and red regions corresponding to positive and negative potentials on the HS mapped over electrostatic potential (Spackman *et al.*, 2008 ▸; Jayatilaka *et al.*, 2005 ▸) shown in Fig. 4 ▸ where the blue regions indicate positive electrostatic potential (hydrogen-bond donors) and the red regions indicate negative electrostatic potential (hydrogen-bond acceptors). The shape-index of the HS is a tool to visualize the π–π stacking by the presence of adjacent red and blue triangles; if there are no adjacent red and/or blue triangles, then there are no π–π inter­actions. Fig. 5 ▸ clearly suggest that there are no π–π inter­actions in (I).

**Figure 6:**
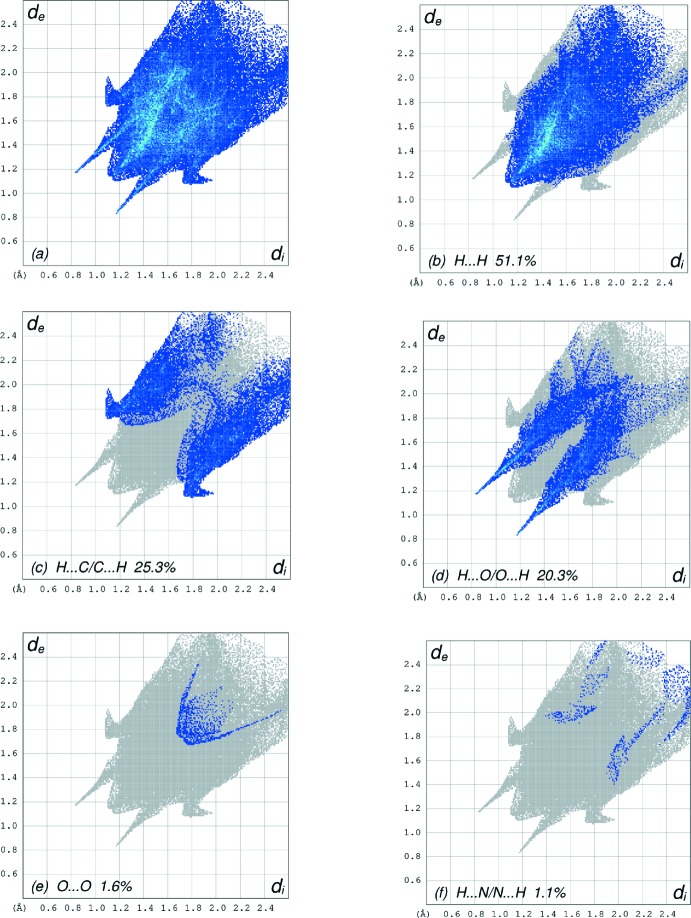
The full two-dimensional fingerprint plots for the title compound, showing (*a*) all inter­actions, and delineated into (*b*) H⋯H, (*c*) H⋯C/C⋯H, (*d*) H⋯O/O⋯H, (*e*) O⋯O and (*f*) H⋯N/N⋯H inter­actions. The *d*_i_ and *d*_e_ values are the closest inter­nal and external distances (in Å) from given points on the Hirshfeld surface.

**Figure 7:**
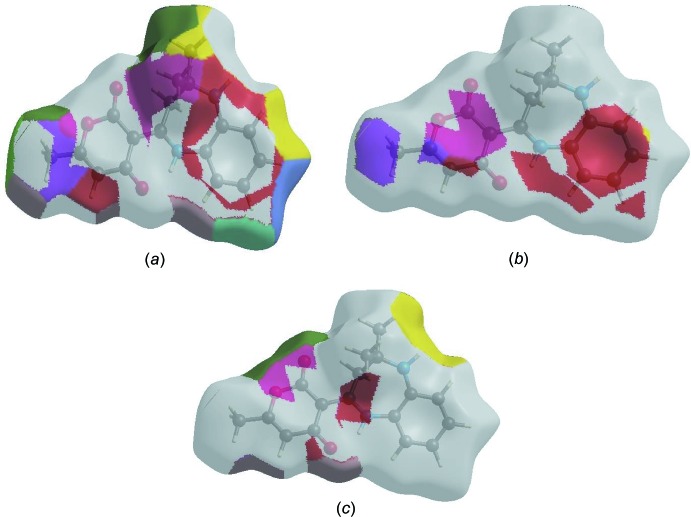
The Hirshfeld surface representations with the function *d*_norm_ plotted onto the surface for (*a*) H⋯H, (*b*) H⋯C/C⋯H and (*c*) H⋯O/O⋯H inter­actions.

The overall two-dimensional fingerprint plot, Fig. 6 ▸ *a*, and those delineated into H⋯H, H⋯C/C⋯H, H⋯O/O⋯H, O⋯O and H⋯N/N⋯H contacts (McKinnon *et al.*, 2007 ▸) are illustrated in Fig. 6 ▸ *b*–*f*, respectively, together with their relative contributions to the Hirshfeld surface. The most important inter­action is H⋯H, contributing 51.1% to the overall crystal packing, which is reflected in Fig. 6 ▸ *b* as widely scattered points of high density due to the large hydrogen content of the mol­ecule. The spike with the tip at *d*_e_ = *d*_i_ = 1.14 Å in Fig. 6 ▸ *b* is due to the short inter­atomic H ⋯ H contacts (Table 2 ▸). In the presence of C—H ⋯ π inter­actions, the pair of wings in the fingerprint plot delineated into H ⋯ C/C ⋯ H contacts with 25.3% contribution to the HS show a nearly symmetrical distribution of points, Fig. 6 ▸ *c*, with the thin edges at d_e_ + d_i_ ∼2.81 Å arising from the H ⋯ C/C ⋯ H contacts (Table 2 ▸). There is a pair of characteristic wings in the fingerprint plot delineated into H⋯O/O⋯H contacts, Fig. 6 ▸ *d:* the 20.3% contribution to the HS arises from the N—H⋯O and C—H⋯O hydrogen bonds (Table 1 ▸) as well as from the H⋯O/O⋯H contacts (Table 2 ▸) and is shown as a pair of spikes with the tips at *d*_e_ + *d*_i_ = 2.00 Å. Finally, the weak O⋯O (Fig. 6 ▸ *e*) and H⋯N/N⋯H (Fig. 6 ▸ *f*) contacts in the structure contribute only 1.6 and 1.1%, respectively, to the HS. The Hirshfeld surface representations with the function *d*_norm_ plotted onto the surface are shown for the H⋯H, H⋯C/C⋯H and H⋯O/O⋯H inter­actions in Fig. 7 ▸ *a*–*c*, respectively.

The Hirshfeld surface analysis confirms the importance of H-atom contacts in establishing the packing. The large number of H⋯H, H⋯C/C⋯H and H⋯O/O⋯H inter­actions suggest that van der Waals inter­actions and hydrogen bonding play the major roles in the crystal packing (Hathwar *et al.*, 2015 ▸).

## Synthesis and crystallization   

A solution of de­hydro­acetic acid (0.168 g, 1 mmol) and *o*-phenyl­enedi­amine (0.108 g, 1 mmol) in ethanol (40 ml) was refluxed for 1 h. After cooling to room temperature, the colourless inter­mediate solid compound, a mono-Schiff base, was obtained in 70% yield. The inter­mediate (0.5 g, 1 mmol) was refluxed in acetone (10 ml) for 1h. After cooling, the crystals formed were filtered and dried (yield: 65%).

## Refinement   

Crystal data, data collection and structure refinement details are summarized in Table 3 ▸. N- and C-bound H atoms were positioned geometrically (N—H = 0.86 Å and C—H = 0.93, 0.97 and 0.96 Å for aromatic, methyl­ene and methyl H atoms, respectively) and constrained to ride on their parent atoms, with *U*_iso_(H) = *kU*_eq_(N, C), where *k* = 1.5 for methyl H atoms and 1.2 for the other H atoms.

## Supplementary Material

Crystal structure: contains datablock(s) I, global. DOI: 10.1107/S2056989019000689/lh5890sup1.cif

Structure factors: contains datablock(s) I. DOI: 10.1107/S2056989019000689/lh5890Isup2.hkl

Supporting information file. DOI: 10.1107/S2056989019000689/lh5890Isup3.cdx

Supporting information file. DOI: 10.1107/S2056989019000689/lh5890Isup4.cml

CCDC reference: 1890950

Additional supporting information:  crystallographic information; 3D view; checkCIF report

## Figures and Tables

**Table 1:** Hydrogen-bond geometry (Å, °) *Cg* is the centroid of the C4–C9 ring.

*D*—H⋯*A*	*D*—H	H⋯*A*	*D*⋯*A*	*D*—H⋯*A*
N1—H1⋯O1	0.86	1.79	2.5351 (18)	143
N2—H2⋯O2^v^	0.86	2.16	2.9990 (16)	166
C2—H2*A*⋯O2	0.97	2.12	2.8628 (17)	132
C5—H5⋯O2^v^	0.93	2.72	3.453 (2)	136
C12—H12⋯O1^i^	0.93	2.45	3.3789 (18)	174
C16—H16*A*⋯O2^iv^	0.96	2.48	3.3646 (18)	154
C2—H2*B*⋯*Cg* ^vi^	0.97	2.63	3.4428 (15)	141

Symmetry codes: (i) 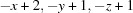; (iv) 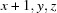; (v) 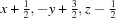; (vi) 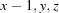.

**Table 2:** Selected interatomic distances (Å)

O1⋯N1	2.5351 (18)	C6⋯H2*B* ^iv^	2.95
O1⋯C12^i^	3.379 (2)	C7⋯H2*B* ^iv^	2.92
O2⋯N2^ii^	2.9990 (16)	C8⋯H2*B* ^iv^	2.94
O2⋯C2	2.8628 (17)	C9⋯H2*B*	2.85
O1⋯H12^i^	2.45	C11⋯H1	2.35
O1⋯H1	1.79	C14⋯H2*A*	2.58
O2⋯H2*A*	2.12	C14⋯H17*C* ^iii^	2.95
O2⋯H2^ii^	2.16	C16⋯O2^iv^	3.3648 (19)
O2⋯H5^ii^	2.72	H1⋯H8	2.24
O2⋯H17*C* ^iii^	2.91	H2⋯H5	2.21
N1⋯N2	3.0698 (17)	H2⋯H17*A*	2.27
N1⋯C16	3.3656 (19)	H2⋯H17*C*	2.59
N2⋯N1	3.0698 (17)	H2*A*⋯H17*B*	2.42
N1⋯H16*A*	2.8152	H2*B*⋯H17*C*	2.57
C4⋯C2^iv^	3.5887 (17)	H5⋯H16*B* ^v^	2.42
C9⋯C2^iv^	3.533 (2)	H5⋯H17*B* ^v^	2.51
C14⋯C17^iii^	3.404 (2)	H12⋯H15*B*	2.45
C1⋯H16*A*	2.62	H12⋯H12^i^	2.55
C4⋯H2*B*	2.79	H16*A*⋯O2^iv^	2.48
C5⋯H2*B* ^iv^	2.99	H16*B*⋯H17*B*	2.52
C5⋯H16*B* ^v^	2.99	H16*C*⋯H17*A*	2.50

Symmetry codes: (i) 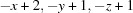; (ii) 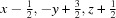; (iii) 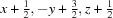; (iv) 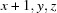; (v) 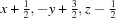.

**Table 3:** Experimental details

Crystal data	
Chemical formula	C_17_H_18_N_2_O_3_
*M*_r_	298.33
Crystal system, space group	Monoclinic, *P*2_1_/*n*
Temperature (K)	296
*a*, *b*, *c* (Å)	5.5373 (1), 24.0197 (4), 11.7815 (3)
β (°)	103.488 (2)
*V* (Å^3^)	1523.77 (6)
*Z*	4
Radiation type	Cu *K*α
μ (mm^−1^)	0.74
Crystal size (mm)	0.42 × 0.38 × 0.16

Data collection	
Diffractometer	Rigaku Oxford Diffraction
Absorption correction	Multi-scan (*CrysAlis PRO*; Rigaku OD 2015 ▸)
*T*_min_, *T*_max_	0.700, 1.000
No. of measured, independent and observed [*I* > 2σ(*I*)] reflections	9860, 2934, 2519
*R*_int_	0.023
(sin θ/λ)_max_ (Å^−1^)	0.614

Refinement	
*R*[*F* ^2^ > 2σ(*F* ^2^)], *wR*(*F* ^2^), *S*	0.040, 0.112, 1.05
No. of reflections	2934
No. of parameters	203
H-atom treatment	H-atom parameters constrained
Δρ_max_, Δρ_min_ (e Å^−3^)	0.20, −0.16

Computer programs: *CrysAlis PRO* (Rigaku OD, 2015 ▸), *SHELXT* (Sheldrick, 2015*b* ▸), *SHELXL2014* (Sheldrick, 2015*a* ▸) and *OLEX2* (Dolomanov *et al.*, 2009 ▸).

## supplementary crystallographic information

### Crystal data

**Table:**

C_17_H_18_N_2_O_3_	*F*(000) = 632
*M**_r_* = 298.33	*D*_x_ = 1.300 Mg m^−^^3^
Monoclinic, *P*2_1_/*n*	Cu *K*α radiation, λ = 1.54184 Å
*a* = 5.5373 (1) Å	Cell parameters from 4280 reflections
*b* = 24.0197 (4) Å	θ = 3.8–71.5°
*c* = 11.7815 (3) Å	µ = 0.74 mm^−^^1^
β = 103.488 (2)°	*T* = 296 K
*V* = 1523.77 (6) Å^3^	Prism, yellow
*Z* = 4	0.42 × 0.38 × 0.16 mm

### Data collection

**Table:**

Rigaku Oxford Diffraction diffractometer	2934 independent reflections
Radiation source: fine-focus sealed X-ray tube, Enhance (Cu) X-ray Source	2519 reflections with *I* > 2σ(*I*)
Graphite monochromator	*R*_int_ = 0.023
Detector resolution: 16.0416 pixels mm^-1^	θ_max_ = 71.3°, θ_min_ = 3.7°
ω scans	*h* = −5→6
Absorption correction: multi-scan (CrysAlis PRO; Rigaku OD 2015)	*k* = −27→29
*T*_min_ = 0.700, *T*_max_ = 1.000	*l* = −14→14
9860 measured reflections	

### Refinement

**Table:**

Refinement on *F*^2^	Hydrogen site location: inferred from neighbouring sites
Least-squares matrix: full	H-atom parameters constrained
*R*[*F*^2^ > 2σ(*F*^2^)] = 0.040	*w* = 1/[σ^2^(*F*_o_^2^) + (0.0578*P*)^2^ + 0.3037*P*] where *P* = (*F*_o_^2^ + 2*F*_c_^2^)/3
*wR*(*F*^2^) = 0.112	(Δ/σ)_max_ = 0.001
*S* = 1.05	Δρ_max_ = 0.20 e Å^−^^3^
2934 reflections	Δρ_min_ = −0.16 e Å^−^^3^
203 parameters	Extinction correction: SHELXL2014 (Sheldrick, 2015a), Fc^*^=kFc[1+0.001xFc^2^λ^3^/sin(2θ)]^-1/4^
0 restraints	Extinction coefficient: 0.0020 (3)
Primary atom site location: dual	

### Special details

**Table:**

Geometry. All esds (except the esd in the dihedral angle between two l.s. planes) are estimated using the full covariance matrix. The cell esds are taken into account individually in the estimation of esds in distances, angles and torsion angles; correlations between esds in cell parameters are only used when they are defined by crystal symmetry. An approximate (isotropic) treatment of cell esds is used for estimating esds involving l.s. planes.

### Fractional atomic coordinates and isotropic or equivalent isotropic displacement parameters (Å^2^)

**Table:**

	*x*	*y*	*z*	*U*_iso_*/*U*_eq_	
O1	0.9607 (2)	0.55040 (5)	0.34659 (11)	0.0620 (4)	
O2	0.3736 (2)	0.69299 (4)	0.35380 (9)	0.0499 (3)	
O3	0.45601 (19)	0.62933 (4)	0.48899 (9)	0.0430 (3)	
N1	0.8514 (2)	0.61406 (5)	0.17042 (11)	0.0406 (3)	
H1	0.9435	0.5909	0.2170	0.049*	
N2	0.7655 (3)	0.71643 (5)	0.01003 (11)	0.0478 (3)	
H2	0.7686	0.7426	−0.0394	0.057*	
C1	0.6776 (2)	0.63806 (5)	0.21295 (12)	0.0350 (3)	
C2	0.5128 (2)	0.67882 (6)	0.13633 (12)	0.0382 (3)	
H2A	0.3856	0.6910	0.1752	0.046*	
H2B	0.4303	0.6602	0.0648	0.046*	
C3	0.6492 (2)	0.73059 (5)	0.10487 (11)	0.0342 (3)	
C4	0.8700 (2)	0.66752 (6)	−0.01123 (12)	0.0377 (3)	
C5	0.9507 (3)	0.66471 (7)	−0.11604 (14)	0.0491 (4)	
H5	0.9191	0.6948	−0.1670	0.059*	
C6	1.0733 (4)	0.61968 (8)	−0.14591 (17)	0.0644 (5)	
H6	1.1245	0.6198	−0.2157	0.077*	
C7	1.1215 (4)	0.57393 (8)	−0.0726 (2)	0.0733 (6)	
H7	1.2073	0.5434	−0.0917	0.088*	
C8	1.0400 (4)	0.57462 (7)	0.02874 (18)	0.0597 (5)	
H8	1.0711	0.5439	0.0780	0.072*	
C9	0.9117 (3)	0.62000 (6)	0.06068 (13)	0.0406 (3)	
C10	0.6538 (2)	0.62144 (5)	0.32672 (12)	0.0355 (3)	
C11	0.7997 (3)	0.57556 (6)	0.38662 (13)	0.0434 (3)	
C12	0.7494 (3)	0.55810 (6)	0.49603 (13)	0.0483 (4)	
H12	0.8365	0.5281	0.5357	0.058*	
C13	0.5820 (3)	0.58377 (6)	0.54124 (13)	0.0429 (3)	
C14	0.4890 (2)	0.65029 (5)	0.38391 (11)	0.0360 (3)	
C15	0.5058 (4)	0.56883 (8)	0.65044 (15)	0.0594 (4)	
H15A	0.5417	0.5993	0.7045	0.089*	
H15B	0.5957	0.5364	0.6845	0.089*	
H15C	0.3309	0.5611	0.6327	0.089*	
C16	0.8401 (3)	0.75272 (6)	0.20995 (13)	0.0459 (4)	
H16A	0.9684	0.7255	0.2348	0.069*	
H16B	0.7605	0.7602	0.2725	0.069*	
H16C	0.9118	0.7864	0.1888	0.069*	
C17	0.4561 (3)	0.77557 (6)	0.05783 (13)	0.0457 (4)	
H17A	0.5355	0.8065	0.0297	0.069*	
H17B	0.3814	0.7880	0.1192	0.069*	
H17C	0.3303	0.7605	−0.0049	0.069*	

### Atomic displacement parameters (Å^2^)

**Table:**

	*U*^11^	*U*^22^	*U*^33^	*U*^12^	*U*^13^	*U*^23^
O1	0.0764 (8)	0.0527 (7)	0.0685 (8)	0.0335 (6)	0.0406 (6)	0.0244 (6)
O2	0.0595 (7)	0.0469 (6)	0.0441 (6)	0.0227 (5)	0.0136 (5)	0.0028 (4)
O3	0.0484 (6)	0.0432 (5)	0.0407 (5)	0.0071 (4)	0.0171 (4)	0.0020 (4)
N1	0.0468 (7)	0.0331 (6)	0.0463 (7)	0.0048 (5)	0.0197 (5)	0.0062 (5)
N2	0.0655 (8)	0.0409 (6)	0.0456 (7)	0.0080 (6)	0.0304 (6)	0.0104 (5)
C1	0.0371 (7)	0.0296 (6)	0.0400 (7)	−0.0031 (5)	0.0122 (5)	0.0007 (5)
C2	0.0335 (6)	0.0443 (7)	0.0373 (7)	−0.0013 (5)	0.0095 (5)	0.0056 (6)
C3	0.0351 (7)	0.0353 (7)	0.0338 (6)	0.0037 (5)	0.0110 (5)	0.0048 (5)
C4	0.0371 (7)	0.0405 (7)	0.0375 (7)	−0.0075 (5)	0.0131 (5)	−0.0052 (5)
C5	0.0563 (9)	0.0541 (9)	0.0424 (8)	−0.0096 (7)	0.0225 (7)	−0.0055 (6)
C6	0.0835 (13)	0.0615 (10)	0.0630 (11)	−0.0134 (9)	0.0472 (10)	−0.0181 (9)
C7	0.0968 (15)	0.0455 (9)	0.0989 (15)	−0.0026 (9)	0.0660 (13)	−0.0179 (9)
C8	0.0756 (12)	0.0344 (7)	0.0830 (12)	−0.0005 (7)	0.0469 (10)	−0.0019 (7)
C9	0.0441 (7)	0.0350 (7)	0.0486 (8)	−0.0060 (5)	0.0223 (6)	−0.0053 (6)
C10	0.0395 (7)	0.0297 (6)	0.0389 (7)	0.0018 (5)	0.0125 (6)	0.0026 (5)
C11	0.0495 (8)	0.0355 (7)	0.0492 (8)	0.0087 (6)	0.0197 (6)	0.0079 (6)
C12	0.0584 (9)	0.0408 (7)	0.0483 (8)	0.0116 (6)	0.0178 (7)	0.0142 (6)
C13	0.0509 (8)	0.0389 (7)	0.0398 (7)	−0.0001 (6)	0.0128 (6)	0.0038 (6)
C14	0.0378 (7)	0.0342 (6)	0.0351 (6)	0.0023 (5)	0.0067 (5)	−0.0004 (5)
C15	0.0751 (11)	0.0606 (10)	0.0477 (9)	0.0014 (8)	0.0251 (8)	0.0077 (7)
C16	0.0445 (8)	0.0412 (8)	0.0490 (8)	0.0001 (6)	0.0048 (6)	−0.0002 (6)
C17	0.0462 (8)	0.0482 (8)	0.0437 (8)	0.0121 (6)	0.0127 (6)	0.0121 (6)

### Geometric parameters (Å, º)

**Table:**

O1—C11	1.2569 (18)	C6—H6	0.9300
O2—C14	1.2167 (17)	C6—C7	1.385 (3)
O3—C13	1.3649 (18)	C7—H7	0.9300
O3—C14	1.3874 (16)	C7—C8	1.372 (3)
N1—H1	0.8600	C8—H8	0.9300
N1—C1	1.3168 (18)	C8—C9	1.400 (2)
N1—C9	1.4158 (18)	C10—C11	1.4487 (18)
N2—H2	0.8600	C10—C14	1.4331 (18)
N2—C3	1.4542 (17)	C11—C12	1.443 (2)
N2—C4	1.3584 (19)	C12—H12	0.9300
C1—C2	1.4907 (18)	C12—C13	1.324 (2)
C1—C10	1.4338 (18)	C13—C15	1.488 (2)
C2—H2A	0.9700	C15—H15A	0.9600
C2—H2B	0.9700	C15—H15B	0.9600
C2—C3	1.5444 (18)	C15—H15C	0.9600
C3—C16	1.524 (2)	C16—H16A	0.9600
C3—C17	1.5301 (18)	C16—H16B	0.9600
C4—C5	1.4094 (19)	C16—H16C	0.9600
C4—C9	1.408 (2)	C17—H17A	0.9600
C5—H5	0.9300	C17—H17B	0.9600
C5—C6	1.366 (2)	C17—H17C	0.9600
			
O1···N1	2.5351 (18)	C6···H2B^iv^	2.95
O1···C12^i^	3.379 (2)	C7···H2B^iv^	2.92
O2···N2^ii^	2.9990 (16)	C8···H2B^iv^	2.94
O2···C2	2.8628 (17)	C9···H2B	2.85
O1···H12^i^	2.45	C11···H1	2.35
O1···H1	1.79	C14···H2A	2.58
O2···H2A	2.12	C14···H17C^iii^	2.95
O2···H2^ii^	2.16	C16···O2^iv^	3.3648 (19)
O2···H5^ii^	2.72	H1···H8	2.24
O2···H17C^iii^	2.91	H2···H5	2.21
N1···N2	3.0698 (17)	H2···H17A	2.27
N1···C16	3.3656 (19)	H2···H17C	2.59
N2···N1	3.0698 (17)	H2A···H17B	2.42
N1···H16A	2.8152	H2B···H17C	2.57
C4···C2^iv^	3.5887 (17)	H5···H16B^v^	2.42
C9···C2^iv^	3.533 (2)	H5···H17B^v^	2.51
C14···C17^iii^	3.404 (2)	H12···H15B	2.45
C1···H16A	2.62	H12···H12^i^	2.55
C4···H2B	2.79	H16A···O2^iv^	2.48
C5···H2B^iv^	2.99	H16B···H17B	2.52
C5···H16B^v^	2.99	H16C···H17A	2.50
			
C13—O3—C14	122.24 (11)	C4—C9—N1	126.21 (12)
C1—N1—H1	114.6	C8—C9—N1	114.18 (14)
C1—N1—C9	130.71 (12)	C8—C9—C4	119.45 (14)
C9—N1—H1	114.6	C1—C10—C11	120.32 (12)
C3—N2—H2	115.5	C14—C10—C1	120.82 (12)
C4—N2—H2	115.5	C14—C10—C11	118.85 (12)
C4—N2—C3	128.91 (11)	O1—C11—C10	123.15 (13)
N1—C1—C2	117.10 (12)	O1—C11—C12	119.80 (13)
N1—C1—C10	117.94 (12)	C12—C11—C10	117.03 (12)
C10—C1—C2	124.90 (12)	C11—C12—H12	119.2
C1—C2—H2A	108.7	C13—C12—C11	121.53 (13)
C1—C2—H2B	108.7	C13—C12—H12	119.2
C1—C2—C3	114.17 (11)	O3—C13—C15	111.40 (13)
H2A—C2—H2B	107.6	C12—C13—O3	121.61 (13)
C3—C2—H2A	108.7	C12—C13—C15	126.99 (14)
C3—C2—H2B	108.7	O2—C14—O3	113.36 (12)
N2—C3—C2	108.97 (11)	O2—C14—C10	128.26 (13)
N2—C3—C16	110.99 (12)	O3—C14—C10	118.35 (11)
N2—C3—C17	106.51 (11)	C13—C15—H15A	109.5
C16—C3—C2	111.80 (11)	C13—C15—H15B	109.5
C16—C3—C17	109.98 (12)	C13—C15—H15C	109.5
C17—C3—C2	108.41 (11)	H15A—C15—H15B	109.5
N2—C4—C5	116.02 (13)	H15A—C15—H15C	109.5
N2—C4—C9	127.38 (12)	H15B—C15—H15C	109.5
C9—C4—C5	116.58 (13)	C3—C16—H16A	109.5
C4—C5—H5	118.6	C3—C16—H16B	109.5
C6—C5—C4	122.80 (16)	C3—C16—H16C	109.5
C6—C5—H5	118.6	H16A—C16—H16B	109.5
C5—C6—H6	119.9	H16A—C16—H16C	109.5
C5—C6—C7	120.20 (15)	H16B—C16—H16C	109.5
C7—C6—H6	119.9	C3—C17—H17A	109.5
C6—C7—H7	120.7	C3—C17—H17B	109.5
C8—C7—C6	118.58 (16)	C3—C17—H17C	109.5
C8—C7—H7	120.7	H17A—C17—H17B	109.5
C7—C8—H8	118.9	H17A—C17—H17C	109.5
C7—C8—C9	122.30 (17)	H17B—C17—H17C	109.5
C9—C8—H8	118.9		
			
C9—N1—C1—C10	177.02 (13)	C1—N1—C9—C8	−157.26 (15)
C9—N1—C1—C2	−0.3 (2)	C1—N1—C9—C4	27.4 (2)
N1—C1—C2—C3	−63.12 (16)	N1—C1—C10—C14	172.65 (12)
C10—C1—C2—C3	119.73 (14)	C2—C1—C10—C14	−10.2 (2)
C4—N2—C3—C16	88.57 (18)	N1—C1—C10—C11	−6.4 (2)
C4—N2—C3—C17	−151.73 (15)	C2—C1—C10—C11	170.76 (13)
C4—N2—C3—C2	−35.0 (2)	C14—C10—C11—O1	−175.06 (15)
C1—C2—C3—N2	80.67 (14)	C1—C10—C11—O1	4.0 (2)
C1—C2—C3—C16	−42.38 (16)	C14—C10—C11—C12	6.4 (2)
C1—C2—C3—C17	−163.79 (12)	C1—C10—C11—C12	−174.60 (13)
C3—N2—C4—C9	−6.6 (3)	O1—C11—C12—C13	179.64 (16)
C3—N2—C4—C5	174.97 (14)	C10—C11—C12—C13	−1.7 (2)
N2—C4—C5—C6	175.76 (16)	C11—C12—C13—O3	−2.6 (3)
C9—C4—C5—C6	−2.8 (2)	C11—C12—C13—C15	177.58 (16)
C4—C5—C6—C7	0.6 (3)	C14—O3—C13—C12	2.2 (2)
C5—C6—C7—C8	1.1 (3)	C14—O3—C13—C15	−177.94 (13)
C6—C7—C8—C9	−0.4 (3)	C13—O3—C14—O2	−175.69 (13)
C7—C8—C9—C4	−1.9 (3)	C13—O3—C14—C10	2.60 (19)
C7—C8—C9—N1	−177.57 (18)	C1—C10—C14—O2	−7.8 (2)
N2—C4—C9—C8	−174.98 (16)	C11—C10—C14—O2	171.19 (14)
C5—C4—C9—C8	3.4 (2)	C1—C10—C14—O3	174.15 (12)
N2—C4—C9—N1	0.1 (2)	C11—C10—C14—O3	−6.81 (19)
C5—C4—C9—N1	178.49 (14)		

Symmetry codes: (i) −*x*+2, −*y*+1, −*z*+1; (ii) *x*−1/2, −*y*+3/2, *z*+1/2; (iii) *x*+1/2, −*y*+3/2, *z*+1/2; (iv) *x*+1, *y*, *z*; (v) *x*+1/2, −*y*+3/2, *z*−1/2.

### Hydrogen-bond geometry (Å, º)

*Cg* is the centroid of the C4–C9 ring.

**Table:**

*D*—H···*A*	*D*—H	H···*A*	*D*···*A*	*D*—H···*A*
N1—H1···O1	0.86	1.79	2.5351 (18)	143
N2—H2···O2^v^	0.86	2.16	2.9990 (16)	166
C2—H2*A*···O2	0.97	2.12	2.8628 (17)	132
C5—H5···O2^v^	0.93	2.72	3.453 (2)	136
C12—H12···O1^i^	0.93	2.45	3.3789 (18)	174
C16—H16*A*···O2^iv^	0.96	2.48	3.3646 (18)	154
C2—H2*B*···*Cg*^vi^	0.97	2.63	3.4428 (15)	141

Symmetry codes: (i) −*x*+2, −*y*+1, −*z*+1; (iv) *x*+1, *y*, *z*; (v) *x*+1/2, −*y*+3/2, *z*−1/2; (vi) *x*−1, *y*, *z*.
